# Reflecting on Palliative Care Integration in Canada: A Qualitative Report

**DOI:** 10.3390/curroncol28040240

**Published:** 2021-07-19

**Authors:** Maryam Qureshi, Maggie C. Robinson, Aynharan Sinnarajah, Srini Chary, Janet M. de Groot, Andrea Feldstain

**Affiliations:** 1 Werklund School of Education, University of Calgary, Calgary, AB T2N 1N4, Canada; 2 Tom Baker Cancer Centre, Alberta Health Services, Calgary, AB T2N 4N2, Canada; maggie.robinson@tcd.ie (M.C.R.); janet.degroot@albertahealthservices.ca (J.M.d.G.); Andrea.Feldstain@albertahealthservices.ca (A.F.); 3 Department of Medicine, Queen’s University, Kingston, ON K7L 3J7, Canada; asinnarajah@lh.ca; 4 Department of Medicine, Lakeridge Health, Ajax, ON L1S 2J4, Canada; 5 Cumming School of Medicine, University of Calgary, Calgary, AB T2N 4N1, Canada; 6 Palliative Medicine, Queens University, Kingston, ON K7L 3J7, Canada; sc260@queensu.ca; 7 Department of Psychiatry, Cumming School of Medicine, University of Calgary, Calgary, AB T2N 2T9, Canada

**Keywords:** palliative care, integration, collaboration, interdisciplinary, palliative care teams, palliative approach, palliative care model

## Abstract

Studies have identified integrated interdisciplinary care as a hallmark of effective palliative care. Although models attempt to show how integration may function, there is little literature available that practically explores how integration is fostered and maintained. In this study we asked palliative care clinicians across Canada to comment on how services are integrated across the healthcare system. This is an analysis of qualitative data from a larger study, wherein clinicians provided written responses regarding their experiences. Content analysis was used to identify response categories. Clinicians (*n* = 14) included physicians, a nurse and a social worker from six provinces. They identified the benefits of formalized relationships and collaboration pathways with other services to streamline referral and consultation. Clinicians perceived a need for better training of residents and primary care physicians in the community and more acceptance, shared understanding, and referrals. Clinicians also described integrating well with oncology departments. Lastly, clinicians considered integration a complex process with departmental, provincial, and national involvement. The needs and strengths identified by the clinicians mirror the qualities of successfully integrated palliative care programs globally and highlight specific areas in policy, education, practice, and research that could benefit those in Canada.

## 1. Introduction

Palliative care has been identified as a priority in healthcare due to the increasing proportion of Canadians who are aging or chronically unwell [1,2]. Well-rounded care can increase quality-of-life (QOL) for people throughout their illness and to provide a peaceful death [3,4]. Similarly, the risks of not meeting patients’ palliative care needs adequately include unnecessary suffering, less satisfaction with care, and heightened distress at the end of life [4,5]. The World Health Organization (WHO) identifies several hallmarks of effective palliative care including a focus on holistic well-being, lifespan approach, and interdisciplinary collaboration [1,6,7,8]. In the present study we honed in on interdisciplinary collaboration and integration with other services by interviewing palliative care clinicians. We focused on Canadian clinicians because Canada has been described as a world leader in palliative care [9,10].

Many hallmarks of palliative care are directly linked to the effectiveness of interdisciplinary team integration. For example, the lifespan approach should allow medical teams to address patients’ symptoms and psychosocial needs throughout their illness rather than only at the end of life [3,11]. However, it is unclear to what extent palliative care teams and peripheral/community medical services use this inclusive approach [12], and Canadian evidence revealed that referrals come late despite a clinician’s understanding of the wider scope of care [13]. In addition, it is unclear to what extent patients understand the evolving role of palliative care [12]. 

Another hallmark of effective palliative care is its focus on QOL and holistic well-being rather than solely on remedying the physical illness [6,7,8,14], which recognizes a patient’s psychological, social and spiritual needs [3,15,16,17,18]. For example, this model recognizes that distress in one area can exacerbate others, such that perceptions of cancer-related pain can increase if a patient is experiencing spiritual distress [3]. For a palliative care team to note and address these nuances, however, it needs to have access to multidisciplinary professionals and an integration framework [14].

The issue of delivering effective palliative care is further complicated by the existence of different models [12,19,20]. Urban areas close to hospitals usually have access to specialist services, consisting of experts who have extensive training in palliative care and can provide consults for complex cases [1]. On the other hand, many communities rely on the palliative approach, which relies on non-specialists with a variety of training backgrounds [1,21], but it is unclear to what extent these services share a common understanding or are able to offer an interdisciplinary approach to patient care. As a result, it is even more pressing for us to understand how palliative teams, community physicians and other community services function such that we can maintain or improve existing integration models.

The literature on this is limited, and as such this research study seeks answers to the question: How are Canadian palliative care services integrated across the healthcare system?

## 2. Materials and Methods

### 2.1. Participants

This study was approved by the Health Research Ethics Board of Alberta—Cancer Control (#HREBA.CC-17-0271). Using purposive and chain sampling, we recruited palliative-care directors from Canadian hospitals and universities and asked them to invite any interested colleagues. In case of a possible dual-relationships within our research team, all communication and raw data were handled by research assistants (authors MR and MQ), who were one-step removed from the profession for confidentiality reasons.

### 2.2. Questionnaire/Interview Guide

Data were analyzed from a larger study that asked participants to respond to 11 open-ended questions. The questions in our interview guide were based on findings from Williams and colleagues’ study of hospice palliative care and the personal interests of the authors [22]. Open-ended questions were emailed to potential participants in a Microsoft Word document, with the option of responding in writing or setting up a verbal interview with a research assistant. In this study we focused on participants’ answers to one qualitative question: How does palliative care integrate with other disciplines in your region?

### 2.3. Procedure

In our first wave of recruitment we specifically searched for directors of clinical and academic palliative care programs in each Canadian province and territory. Categories in our data did not reach saturation; thus, in our second wave we recruited from a wider pool through the Canadian Society for Palliative Care Physicians (CSPCP)’s members mailing list. Members of the CSPCP were sent the same invitation, questionnaire, and request for chain sampling; however. to bolster participation we included materials in English and in French.

### 2.4. Data Analysis

To analyze the content of qualitative responses we followed the content analysis procedure of Vaismoradi et al. [23], which came from a positivist ontology and epistemology; in other words, the participants’ accounts are assumed to be truthful indices of reality [24]. In addition, it considered the relevance and frequency of responses and was more descriptive and interpretive. This method was deemed appropriate due to the research question and short nature of written responses. Our analysis procedure was to develop familiarity with the transcripts through re-reading participants’ written accounts [23,25] and then to use open coding based on manifest (directly observable) content. Categories were generated from a large list of codes, which involved negotiating common threads, subsuming some codes under others, and creating new titles to describe the contents of emerging categories. (See Appendix A for a description on how rigour was maintained during qualitative analysis through credibility, dependability, and transferability [26,27].

## 3. Results

Participants (*n* = 87) were contacted in the first wave, and an unknown number were contacted in the second through an email list of secondary organizations. We obtained 17 responses but opted not to include three due to incomplete data and as a result our analysis was based on 14 complete responses. Participants came from six provinces and a variety of clinical settings and careers, with the largest groups being Ontario physicians, hospital workers, and clinicians. In addition, 12 participants provided written responses and two gave telephone interviews. It should be noted that although participants were sought from all provinces and territories, there were several provinces and all territories did not respond (Table 1).

In terms of qualitative findings, we identified four categories that outlined how palliative care integrated with other healthcare services (see Table 2).

### 3.1. Integration through Relationships

Many participants discussed that establishing relationships with other physicians, teams, or specialties was integral for palliative care collaboration. These relationships were described as informal, formal, or transformed from informal to formal.

For some participants, integration was a function of informal relationships. They described this as building goodwill with other professionals, hospital departments, provincial teams or specialists and community clinics. For example when asked about integration, one participant said, “A lot is based on goodwill and establishing networks of communication”. A participant however, suggested that these relationships were highly variable, not standardized, and stated a wish for clearer processes on how to integrate and maintain ties with various programs.

On the other hand, for some participants integration concerned a formal relationship between palliative care and other services in their institutions, such as specific standardized procedures for when and how to involve palliative care. For example, one participant explained:

“It depends on the service: in pediatric oncology, for example, the [pediatric palliative care] team meets with all newly diagnosed patients or families. Many patients will only meet the team once, but at least all know who is on the team and who to ask for in the future as need be.”

To bring these ideas together, a few participants described a bridging process from informal to formal relationships between palliative care physicians and other health care services. For example, a hospice care clinician said that integrative collaborations usually start as informal relationships and over time become formally established in institutional service agreements, which leads to higher quality of care. Two other participants shared how institutional procedures that became formalized were helpful, for example: having one central nurse who decides when to involve specific palliative care team members in community clinics. In addition, collaborating with certain specialties through regular case rounds, note sharing and discussions were helpful. As one participant described:

“From our hospice perspective, I would say that corridors of services are established first informally, between healthcare professionals sharing similar patient populations. Then formal inter-establishment service agreements are elaborated and put into motion, facilitating the inter-agency collaboration, and supporting the fact that it no longer relies on the goodwill of one or two persons.”

### 3.2. Integration through Research and Educational Activities

Several participants discussed integration with other health services through palliative care research and educational activities. For these, collaboration and integration not only hinged on establishing relationships for practice and referral but also for research and dissemination. Some described this in the form of academic and research collaboration between different services and providers (e.g., hospice and hospital, or hospital and primary care) to streamline and determine the best care practices. Others mentioned collaborating across services at the dissemination stage and providing training, conferences and workshops on palliative care models. For example, for academic participants this meant promoting research in their university or involving students and residents in education. One participant described research dissemination training and psycho-education within patient organizations and academia and hospitals:

“I try to integrate as much as possible with training students […] I also take every opportunity when invited to go to do talks, but again that’s because people in the community have said ‘oh, I need to know a bit more about this… who can we get to come and give us a talk?’ and sometimes they happen to contact me.”

Although the benefits of research collaboration and dissemination across medical disciplines were clear, participants also noted that those professionals “are the ones driving their learning needs […] there’s not much that we can do to push it out, they have to pull it in” indicating the importance of personal investment in order for collaboration, research, and educational initiatives to function.

### 3.3. Experiences and Perceptions of Integration with Different Disciplines

Participants described varied experiences and perceptions of collaboration across medical specializations, primary amongst these were family medicine and oncology.

Some perceived that collaboration with family physicians was important for better integrated palliative care. One mentioned that for smaller medical centers or rural areas, palliative care is provided through family medicine; thus, it is important for family physicians to be well equipped to deliver palliative care. Participants’ collaboration experience ranged from excellent to disappointing. As one participant said, “We have pockets of excellence; we have some really good people out there, but the level of competence is vast and variable in family medicine offices”. Furthermore, one clinician felt that few family physicians had the training or comfort level to provide adequate symptom management. Another wished that family physicians engaged with palliative care teams earlier and that increased training and education would lead to better relationships and integration.

In discussions about collaboration with other medical professionals, several participants specifically mentioned oncology as integrating well with palliative care or said that they had a good working relationship with oncologists. Although our questions centered around integration with any health services, no other specialty was discussed as frequently or as positively.

### 3.4. Levels of Integration

**Figure 1:**
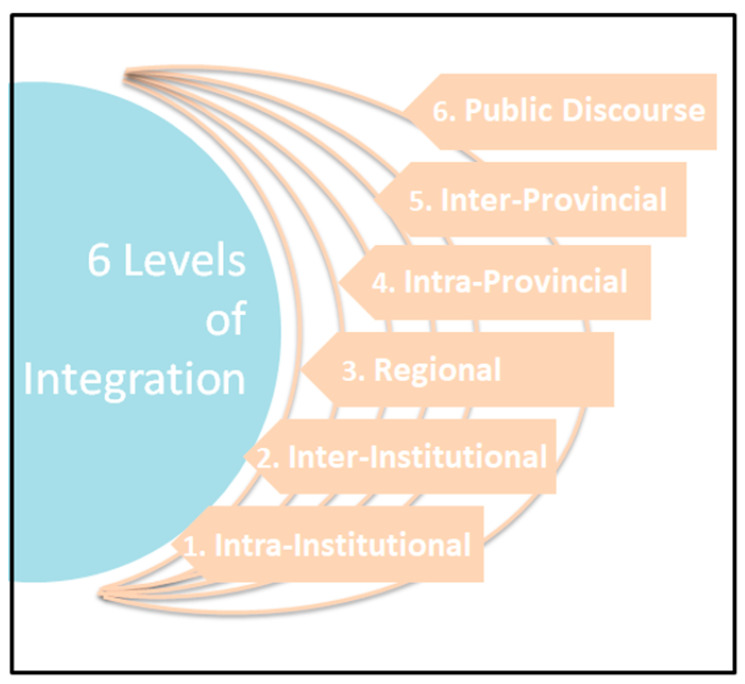
6 Levels of Integration in Palliative Care.

The categories above describe how integration may occur, including areas of success and areas of further development; however, it is also important to consider how clinicians conceptualize integration. Most participants made reference to different levels, ranging from integration within a hospital department, to provincial and national integration strategies. We explored six levels of integration as conceptualized by these participants (Figure 1).

Many participants discussed the first level (intra-institutional): interprofessional teams, departmental meetings, or sharing case notes with different professionals in their department. This includedinter-departmental integration, such as collaborating with different departmental specialists in the same institution or hospital. At the second level (inter-institutional), some participants described cross-institutional integration which took on several forms. One participant mentioned collaboration between a hospice and hospital, and many described collaboration between community primary care and hospital specialized services. At the third level (regional), a few participants described integration between cities, rural or urban areas (e.g., the Greater Toronto Area), or health regions. At the fourth level, participants conceptualized integration as a provincial effort, and some specifically spoke of intra-provincial collaboration strategies. At the fifth level, participants spoke about collaboration across provinces or national strategies. At the sixth and highest level (national, medical and community), participants discussed the need to change the public discourse about palliative care by educating both physicians and patients. These six levels may be envisioned as concentric circles that are all necessary for effective palliative care integration.

## 4. Discussion

Although integration models exist, there is a lack of data concerning how Canadian palliative care services integrate across different health care systems. To explore this question, we gathered qualitative data from fourteen palliative care professionals across Canada, and analyzed clinicians’ qualitative responses. Clinicians identified the benefits of formalized relationships and collaborative procedures with other medical service providers to make referral and consultation decisions easier. Those who were palliative care specialists also identified the need for improved education of trainees, family physicians, or other clinicians in the community who may be missing opportunities for referral to palliative care services. Clinicians also mentioned working very closely and integrating well with oncology departments. Lastly, clinicians had unique definitions of integration and considered multiple levels of integration ranging from intra-departmental to national.

One of the striking observations of our results was that clinicians mostly spoke of integration in relation to other physicians rather than to a diverse range of medical services, which is necessary to meet the psychological, social, and spiritual needs of patients [6,7,8,28]. When asked how palliative care integrated with other services, most of the clinicians mentioned that they made efforts to integrate personally rather than with the help of institution-level procedures. If the majority of collaborative relationships were established by participants individually, this raises the question of whether the onus should be on clinicians to do this. On the one hand, it may be that front-line clinicians are in the best position to establish them because personal relationships lead to stronger institutional working alliances between services. On the other hand, several studies have outlined how formalized referral pathways and automatic triggers for referrals, in contrast to physician-led referrals, reduce patient symptom burden [29,30,31]. Establishing standardized processes within a regional healthcare system may also make it easier for all clinicians in primary care to access palliative care services.

Several participants highlighted that their integration with family physicians varied from excellent to those that could be improved through further training. Past Canadian in surveys showed that 84% of physicians worked in palliative care part-time (7 h per week), and worked as family physicians or in other clinical capacities in the community the rest of the time [32]. This need for family physician acceptance and education in palliative care seems not to have gone unnoticed: 30% of medical students in Canada are now required to complete rotations in palliative care, whereas in the past only 12% were [33,34,35,36]. In addition, 58% of family medicine residents also complete rotations in palliative care [35]. However, on the research side, there remain few faculty positions in Canadian universities focused on palliative care research [33,34,36].

Our participants’ frequent reports of collaboration with oncology departments follows earlier trends in the literature showing that oncology patients make up the majority of palliative care consults [33]. This is explained by the large number of patients diagnosed with cancer and the history of close collaboration between palliative care and oncology. Perhaps the history and stigma of palliative care makes it difficult for other doctors to have these existing relationships or make referrals earlier in an illness, or for patients to accept palliative care referrals.

Upon exploring inter-provincial collaboration, some of our participants noted how, effective palliative care delivery and integration of services may also be complicated by the types of service models (e.g., specialized palliative care teams and palliative approach to care across health disciplines). According to several studies, palliative care is delivered differently depending on location [9,10,32]. Accordingly, we did not have representation from several provinces and could not even find contacts for palliative care services in any of the Canadian territories. We also had one participant who reported providing palliative care in another province for want of other providers. It is of course ideal to have collaboration and integration although it is unrealistic where basic palliative medicine is sparsely available or not at all.

### 4.1. Limitations

Although our study was qualitative, all of our participants except two chose to respond in writing rather than arrange a phone call. Written qualitative data may be convenient for participants but reseachers cannot clarify vague entries or be prompted for further details, as in interviews. Moreover, participants’ responses varied from sentences to paragraphs. Codes and categories emerging from each participants’ responses were not always repeated and we likely did not reach saturation. Future research would benefit from conducting in-depth qualitative interviews to further describe the details of integration, including success stories and recommendations for improvement. In addition, it is important for readers to consider the demographics of our participants and decide on the level of transferability to different contexts. The perspectives in this paper encompassed only some provinces, and mainly the perspective of physicians, and it may not be appropriate to apply the responses of only 14 participants to all palliative care contexts across Canada.

### 4.2. Implications for Future Practice, Policy, and Research

It appears that effective integration with palliative care and other medical services in may hinge on our definition of integration, as well as which models we used to conceptualize integration.

Many of our findings fell into line with recent systematic reviews and global reports of successfully integrated programs, which also provided a definition of integration [37,38]. For example, the systematic review of Hui et al. (2015) identified indicators of integration between oncology and palliative care including 13 clinical, 8 educational, 4 research, and 9 administrative aspects [37,39]. These overarching categories also encompassed ideas that fell into our six levels of integration. In addition, all of our findings fell within these categories, indicating their usefulness for research to assess levels of interdisciplinary palliative integration [37].

Lastly, our participants highlighted how contextually diverse their experiences were across provinces and institutions; thus, further research may benefit from working on a smaller scale to identify key ingredients for individual settings and targeted improvement. A key consideration for researchers and program developers is which models to use to conceptualize integration. Hui and Bruera (2015) discussed integration in the context of a time-based, provider-based, issue-based or system-based model [40]. Each clinical or conceptual model may reveal different necessary interactions between teams based on the environment.

## 5. Conclusions

In conclusion, our sample of Canadian clinicians seemed highly invested in implementing integrated palliative care. They were able to identify several strategies they currently use, and had ideas for how to streamline integration further. That being said, they conceptualized integration in very different ways and spoke almost exclusively about integration with other physicians rather than a variety of service providers. Our discussion of strengths and areas of growth in this study, and in the literature, provide many areas for future practice and research, for example: formalized protocols and relationship networks, a commitment to long-term education and support of primary care physicians, policy change, and acceptance from senior health management. It may be that working within institutions or health regions to determine site-specific solutions for palliative integration is needed.

## Figures and Tables

**Table 1:** Participant Demographic Characteristics.

Demographic Characteristic	Number of Participants
Location	
Alberta	2
British Columbia	2
Ontario	5
Quebec	2
New Brunswick	1
Nova Scotia	2
Clinical Setting	
Hospital (Pediatric or Adult)	7
Specialized Palliative Care Unit	1
Cancer Care	3
Long-term Care	1
Hospice	3
Pediatric Hospice	3
Home Care	2
Academia	1
Management	1
Mixed Urban/Rural Setting	1
Undisclosed	1
Career Stream	
Physician	12
Nurse	1
Social Worker	1

**Table 2:** Qualitative Results Categories.

Categories	Subcategories	Number of Participants Endorsed
Integration through relationships (*n* = 11)	Informal relationships	6
	Formal relationships	2
	Bridge from informal to formal	3
Integration through research and educational activities (*n* = 6)		
Experiences and perceptions of integration with different specialties (*n* = 10)	Oncology	6
	Family Medicine	4
Levels of integration (*n* = 12)	Intra-institutional	8
	Inter-institutional	5
	Regional	4
	Intraprovincial	3
	Interprovincial	1
	Public Discourse	2

## Data Availability

For the confidentiality of participants, qualitative raw data will not be made available.

## Appendix A

Establishing trustworthiness and rigour in qualitative research often involves the consideration of credibility, dependability, and transferability [26,27]. Credibility involves the fit between participants’ responses and the researchers’ representation of them. We maintained credibility throughout the analysis process through peer debriefing, and three team members (MQ, MR, AF) discussing the fit of transcripts with codes, and transcripts with categories, as well as making methodological decisions.

Dependability may be ensured by describing the research process accurately and clearly. We kept an audit trail of the research and analysis process, documenting steps taken and the rationale for methodological and theoretical decisions made as a team. In addition, the researcher primarily involved in analysis (MQ) kept a reflexivity journal. Transferability in qualitative research involves the transfer of findings from one case to another. To help audiences decide if these results are transferable to their circumstances, the researcher is responsible for providing thick descriptions of the participants’ contexts, within the bounds of ethical considerations. Due to confidentiality of certain demographic information ensured in consent, we were not able to release all demographic characteristics but attempted to include as much as ethically possible.
